# MaaT013 fecal microbiotherapy modulates key gastro-intestinal and immunological processes involved in GvHD pathogenesis *in vitro* and *in vivo*

**DOI:** 10.3389/fmicb.2026.1920614

**Published:** 2026-09-14

**Authors:** Bastien Laperrousaz, Julie Reygner, Margaux de Seilhac, Antoine Sabourin, Cassandra Bompard, Cyrielle Gasc, Emmanuel Prestat, Benoît Levast, Charlotte Petitjean, Sophie Declomesnil, Emmanuelle Godefroy, Carole Schwintner, Nathalie Corvaïa, Robert R. Jenq, Ernst Holler

**Affiliations:** 1 MaaT Pharma, Lyon, France; 2 INSERM, CNRS, Immunology and New Concepts in ImmunoTherapy (INCIT), University of Angers, Nantes University, Nantes, France; 3 Genoway, Lyon, France; 4 Department of Hematology & Hematopoietic Cell Transplantation, City of Hope, Duarte, CA, United States; 5 Department of Internal Medicine III (Haematology/Oncology), University Hospital Regensburg, Regensburg, Germany

**Keywords:** acute graft-vs.-host disease (aGVHD), allogeneic hematopoietic cell transplantation (allo-HCT), dendritic cells, dysbiosis, engraftment, fecal microbiotherapy, gut barrier, inflammation

## Abstract

**Background:**

Acute graft-vs.-host disease (aGvHD) is a life-threatening complication of allogeneic hematopoietic cell transplantation (allo-HCT), where allogeneic donor T cells recognize recipient tissues as foreign and damage them. Gut microbiota disruption following allo-HCT has been associated with increased aGvHD incidence, severity, and mortality. Growing clinical evidence suggests the efficacy of fecal microbiotherapy to restore a healthy and diverse gut microbiota and to treat aGvHD. MaaT013 (Xervyteg^®^), a standardized allogeneic fecal microbiotherapy derived from pooled healthy human fecal microbiota, has demonstrated efficacy in mitigating steroid-refractory aGvHD in the Phase 2 HERACLES trial, and is currently being evaluated in the pivotal ARES trial in ruxolitinib-resistant aGvHD with gastrointestinal involvement.

**Methods:**

Here, multi-omics analyses across complementary *in vitro* and *in vivo* models suggest that MaaT013 modulates biological processes involved in aGvHD pathogenesis.

**Results:**

First, MaaT013-derived metabolites prevented inflammation-induced gut barrier alteration in an *in vitro* leaky gut model. Second, MaaT013 stimulated dendritic cell-mediated Tregs amplification and subsequent release of IL-10 and TGF-b *in vitro*. Third, beneficial bacteria from MaaT013 engrafted in the gut of germ-free mice, producing anti-inflammatory metabolites and restoring healthy gut physiology through immune rebalancing. Finally, MaaT013 delayed the appearance of aGvHD symptoms in a specific-pathogen free xenogeneic mouse model of aGvHD.

**Discussion:**

Together, these results provide exploratory, multi-model evidence of MaaT013's potential to mitigate aGvHD by restoring microbial diversity, gut integrity, and immune homeostasis.

## Introduction

Despite the success of allogenic hematopoietic cell transplantation (allo-HCT) for the treatment of hematopoietic malignancies, its efficacy is limited by a major complication: graft-vs.-host disease (GvHD). Acute GvHD (aGvHD) is a serious and life-threatening disease caused by the allo-activation of immunocompetent T cells from the donor graft, which recognizes the recipient's tissues antigens as foreign and initiates an immune response against them (Malard et al., 2023a). Approximately 30% to 60% of allo-HCT recipients develop aGvHD within the first 100 days post-transplant, which has a significant impact on morbidity and mortality (Ferrara et al., 2009; MacMillan et al., 2012; Castilla-Llorente et al., 2014; Holtan et al., 2022). Although aGvHD affects different organs such as the skin or the liver, the gastrointestinal (GI) tract is considered the most serious aGvHD manifestation, given its association with high rates of mortality (Ferrara et al., 2011; MacMillan et al., 2012). Indeed, by damaging the structure of the gut barrier, the endocrine secretion of antimicrobial peptides, and the diversity of gut microbiota providing competitive exclusion against pathogens, aGvHD makes patients more vulnerable to bacteremia (Eriguchi et al., 2012). Around half of patients with aGvHD respond to first-line treatment with corticosteroids (Mohty et al., 2020), and steroid-refractory aGvHD patients have a 1-year survival rate of 25% (Castilla-Llorente et al., 2014). Ruxolitinib, a selective JAK1/2 inhibitor, is currently recommended as a second-line treatment for steroid-refractory or steroid-dependent aGvHD, offering significant clinical benefits (Mohty et al., 2025). However, therapeutic resistance remains a challenge; 48% of subjects did not show overall clinical response to ruxolitinib at Day 28 in the REACH2 trial (Zeiser et al., 2020), highlighting the need for development of novel therapies for this patient population.

Restoration of the gut microbiota is a promising therapeutic target for acute GvHD that may help to address this gap. The conditioning regimen administered before allo-HCT, essential to reduce tumor burden and to provide sufficient immunoablation to prevent graft rejection, is known to induce profound damage to host tissues and significantly disrupt the gut microbiota. High-dose chemotherapy, total-body irradiation, prophylactic and broad-spectrum therapeutic antibiotic treatment of neutropenic fever and infections cause significant intestinal dysbiosis characterized by a loss of microbial diversity and significant alterations in the GI microbial composition (Vliet et al., 2009; Zwielehner et al., 2011; Montassier et al., 2014; Taur et al., 2014; Holler et al., 2014). Gut microbiota disruption following allo-HCT has been associated with increased aGvHD incidence, severity, and mortality (Eriguchi et al., 2012; Taur et al., 2014; Jenq et al., 2015; Weber et al., 2017; Peled et al., 2020). In particular, gut microbiota diversity has been described as an independent predictor of mortality in allo-HCT recipients (Taur et al., 2014) as well as in aGvHD patients (Jenq et al., 2015). Gut microbiota plays a critical role in human health by assisting digestion and releasing key microbial-derived metabolites such as short-chain fatty acids (SCFAs) or tryptophan-derived indoles. These metabolites support the structure and function of the gut barrier, preventing unwanted substances from entering the bloodstream. A healthy gut microbiota also prevents colonization by harmful pathogens by occupying ecological niches and producing anti-microbial peptides (AMPs; Orberg et al., 2024). Finally, by modulating the development of Peyer's patches, the production of IgA, and the differentiation of regulatory T cells (Tregs; Nakamura et al., 2015), gut microbiota maintains the balance between immune exclusion of harmful pathogens and immune tolerance of commensal microorganisms, thereby shaping local and systemic immunity.

These observations prompted studies testing the efficacy of fecal microbiotherapies to restore a healthy and diverse gut microbiota and to treat or prevent aGvHD with gastro-intestinal (GI) involvement. Growing clinical evidence now supports the efficacy of fecal microbiotherapy to improve aGvHD outcome (Kakihana et al., 2016; Spindelboeck et al., 2017; Qi et al., 2018; Shouval et al., 2018, 2019; Zhong et al., 2019; Biernat et al., 2020; Chakrabartty et al., 2020; Goloshchapov et al., 2020; Mao et al., 2020; Bilinski et al., 2021; Biliński et al., 2021; Russia et al., 2021; Merli et al., 2022). These improvements include reduced GI symptoms, better overall gut health, and decreased reliance on immunosuppressive medications. Notably, fecal microbiotherapy studies highlight the promising synergistic potential of combining ruxolitinib or other immunosuppressive agents with fecal microbiotherapy (Biliński et al., 2021; Liu et al., 2022). Thus, fecal microbiotherapy emerges as a promising therapeutic option for patients with aGvHD, particularly those who have not responded to conventional treatments (Kim et al., 2023; Weber et al., 2024).

However, the intrinsic variability in taxonomic composition of donor-derived fecal microbiotherapies limits the translatability of these studies, indicating the need for a more standardized pharmaceutical approach. Fecal microbiotherapies derived from pools of multiple donors have shown superior clinical benefit in ulcerative colitis and greater reproducibility in key functional properties in preclinical models (Levast et al., 2023; Laperrousaz et al., 2024; Reygner et al., 2024; Gefen et al., 2025). MaaT013 (Xervyteg^®^) is a standardized allogeneic fecal microbiotherapy derived from pooled healthy human fecal microbiota and administered via rectal enema. MaaT013 has demonstrated clinical efficacy in second line steroid-resistant GI aGvHD patients in the phase 2 HERACLES study (Malard et al., 2023b) and is currently being investigated in ruxolitinib-resistant third-line patients in the pivotal ARES study (Malard et al., 2025). MaaT013 was safe in this highly immunocompromised patient population.

Because of the multifactorial aspect of the disease, the pleiotropic functions of gut microbiota and the complexity of microbiome-host interactions, elucidating the exact mechanism of action of fecal microbiotherapies in aGvHD is challenging. In particular, how these therapies impact key processes involved in aGvHD pathogenesis, such as microbial dysbiosis, intestinal barrier disruption, and inflammation, remains to be fully understood. The scope of this study was to generate mechanistic insights into the biological activity of MaaT013 through exploratory preclinical investigations, with the aim of informing hypotheses regarding its potential mechanism of action in aGvHD. The study was designed to characterize the biological processes associated with MaaT013 microbial ecosystem activity and was not intended to assign causal roles to individual microbial strains or specific metabolites, nor to fully disentangle the respective contributions of live microbial engraftment and metabolite-mediated effects. Accordingly, *in vitro* and *in vivo* analyses of MaaT013 across complementary models provide an exploratory assessment of its biological activity. Specifically, we evaluated (i) the effects of MaaT013-derived metabolites on gut barrier integrity using a leaky gut model, (ii) the capacity of MaaT013 to modulate innate immune signaling and dendritic cell function, thereby promoting regulatory T cell responses, and (iii) the engraftment, metabolic activity, and host responses to MaaT013 in germ-free and specific-pathogen free (SPF) NCG mice humanized with human PBMCs. Together, these approaches provide a comprehensive, multi-model evaluation of the biological processes associated with MaaT013 activity and deliver mechanistic insights into how microbiota-based therapies may contribute to the regulation of aGvHD pathogenesis.

## Material and methods

### Test substance

MaaT013 (Xervyteg^®^) is a standardized and high-richness microbiotherapy product manufactured in a European cGMP production facility by pooling fecal material from 4 to 8 strictly vetted, healthy donors, using a proprietary cryoprotectant preserving a high concentration of viable cells in the drug substance (minimum of 5.1 × 10^11^ viable bacteria per 150 mL). All healthy donors provided informed consent, and the protocol was approved by ethics committee CPP 2019-A01963-54. Each individual donation was processed following an industrialized process including the pooling step and the addition of the proprietary cryoprotectant. All products were stored as frozen suspensions at −80 °C until use.

### Caco-2/THP1-blue co-culture model

#### Healthy gut test

Permeability assays were performed by Prodigest (Ghent, Belgium). Caco-2 cells (HTB-37, ATCC) were seeded at 100,000 cells per insert (200 μL apical; 700 μL basolateral) in 24-well permeable supports (cellQart, 0.4 μm, Sabeu) and cultured for 14 days with tri-weekly medium changes until a differentiated monolayer with stable transepithelial electrical resistance (TEER) was obtained, following standard epithelial culture procedures (Sapp et al., 2024). TEER was used as a measure of barrier integrity. Cells were maintained in DMEM supplemented with glucose, glutamine, HEPES, and 20% FBS. TEER at baseline (0 h) was measured using a Millicell^®^ ERS-2 meter (Merck). MaaT013 samples were thawed for 5 min at 37 °C, diluted 1:2 in complete Caco-2 medium, centrifuged (10 min, 10,000 g), and sterile-filtered (0.22 μm). The apical compartment received either MaaT013 supernatant (20%), cryoprotectant control, or complete medium. TEER was measured again after 24 h, and values were normalized to the corresponding 0 h measurement. All conditions were performed in triplicate. Cells were incubated at 37 °C in a humidified 5% CO_2_ atmosphere.

#### Leaky gut test

THP1-Blue™ cells (InvivoGen) were maintained in RPMI-1640 supplemented with glucose, glutamine, HEPES, sodium pyruvate, and 10% FBS. These cells contain an NF-κB–inducible SEAP. Cells were seeded at 500,000 cells per well (24-well plates) and differentiated for 48 h with PMA (100 ng/mL). Coculture procedures followed established epithelial–immune interface models (Sapp et al., 2024). Prior to coculture, TEER of the Caco-2 inserts was recorded (0 h). Inserts were then transferred onto PMA-differentiated THP1-Blue™ cells. The apical chamber was filled with centrifuged and sterile-filtered MaaT013 supernatant (20%) or cryoprotectant control; an additional negative control consisted of complete Caco-2 medium (altered condition). Sodium butyrate (12 mM) served as a positive control for barrier restoration. After 24 h, TEER was measured again (24 h), and basolateral medium was collected for CXCL10, MCP-1, IL-6 and IL-10 quantification. All treatments were performed in triplicate. Cultures were maintained at 37 °C with 5% CO_2_.

### TLR/NOD activation assay

The activation assay was performed by INRAE (Jouy en Josas, France). HEK-Blue reporter cell lines (InvivoGen) were used to assess Toll-like receptor (TLR) and Nucleotid-binding Oligomerization Domain proteins (NOD) innate immune receptors activation as previously described (Gilbert et al., 2013; Hovingh et al., 2017). HEK-Blue cells were submitted to routine selection pressure using 100 mg/mL of zeocin for HEK-Blue NULL1 and NULL2, 100 mg/mL of zeocin and 10 mg/mL of blasticidin for HEK-Blue hTLR5, hNOD1 and hNOD2; 1x of HEK-Blue selection mix for HEK-Blue hTLR4 and hTLR2 cell lines. For TLR and NOD stimulation assays, HEK-Blue reporter cells were seeded at a density of 2.5 × 10^4^ cells/well (96-well plate) in RPMI-1640 medium complemented with 10% FBS, 1% L-glutamine and 1% penicillin/streptomycin and incubated 24 h at 37 °C, 5% CO_2_. After 24 h, MaaT013 test substances were thawed on ice and diluted 1:5 in cell culture medium. After centrifugation at 10,000g for 10 min at 4 °C, the supernatants were collected, filtered at 0.22 μm and added to the wells (final concentration 1/100). HEK-Blue reporter cells were incubated for 24 h at 37 °C, 5% CO_2_. In addition, specific TLR and NOD ligands were used as positive controls: Pam3CSK4 (TLR2 ligand) at 100 ng/mL, LPS-EB (TLR4 ligand) at 100 ng/mL, RecFla-ST (TLR5 ligand) at 10 ng/mL, C12-IE-DAP (NOD1 ligand) at 100 ng/mL and L18-MDP (NOD2 ligand) at 100 ng/mL. TNFα pan-positive control was used at 10 ng/mL. After 24 h of incubation, 20 μL of supernatant was collected and receptor activation was assessed using QUANTI-blue detection kit following manufacturer's instructions (InvivoGen). The optical densities (OD) were measured by a spectrophotometer at 655 nm. Fold changes were calculated as follows: OD treatment / OD control medium. All treatments were done in triplicates. Results are expressed as the mean of 2 independent experiments.

### moDC activation assay

Blood samples were collected into platelet-leukocyte concentrates from healthy volunteers at the Nantes Blood Center (EFS, CPDL-PLER-2021-109). PBMCs from 6 different healthy donors were isolated by Ficoll gradient centrifugation (lymphocytes separation medium, EurobioScientific). Monocytes were purified from PBMCs using CD14 microbeads (Miltenyi Biotec) and were differentiated into monocyte-derived dendritic cells (mo-DCs) by a 5- to 6-day culture with 1,000 IU/mL rhGM-CSF (recombinant human Granulocyte-Macrophage Colony-Stimulating Factor) and 300 IU/mL rhIL-4 (CellGenix). Non-adherent immature DCs (iDCs) were harvested at day 5–6 and cultured with or without heat-inactivated (3 cycles of freezing (5 min on ice)/thawing (5 min at 95 °C)) MaaT013 bacteria (ratio 1 DC:25 bacteria; 5 batches) or cryoprotectant as control for 48 h.

### Treg amplification by autologous monocytes assay

Autologous CD4^+^ T cells (comprising CD4^+^ CD25^high^ CD127^low^ Tregs) and CD14+ monocytes were sorted from 5 different healthy donors' PBMCs using magnetic beads (Miltenyi Biotec). CD4^+^ CD25^high^ CD127^low^ Tregs and single positive CD4^+^ T cells were then sorted by flow cytometry and stained with 1 μM violet proliferation dye (VPD, BD Biosciences) before being co-cultured with autologous CD14^+^ monocytes (ratio 1:1) previously loaded overnight with heat-inactivated MaaT013 bacteria (ratio 1 monocyte:5 bacteria; 3 batches), in the presence of low dose rhIL-2 (20 IU/mL; Proleukine, Novartis). Five days later, sorted CD4^+^ CD25^high^ CD127^low^ Treg and single positive CD4^+^ T cell proliferation was measured through VDP dilution analysis by flow cytometry.

### Flow cytometry

Cell surface staining was performed for 40 min at 4 °C in Phosphate Buffered Saline (PBS; PanReac AppliChem)/0.1% Bovin Serum Albumin (BSA; Sigma Aldrich) with antibodies targeting the following markers: CD14 (Biolegend, M5E2 clone), CD11c (Biolegend, Bu15 clone), CD83 (Biolegend, HB15e clone), CD80 (Biolegend, 2D10 clone), CD86 (BD Biosciences, 2331 clone), CD40 (Biolegend, 5C3 clone), CD39 (BD Biosciences, TU66 clone), PD-L1 (BD Biosciences, MIH1 clone) and Class II HLA (DP, DQ, DR; Invitrogen, WR18 clone), and for Treg assay CD3 (Biolegend, UCHT1 clone), CD4 (Beckman Coulter, 13B8.2 clone), CD25 (BD Biosciences, M-A251 clone), CD127 (BD Biosciences, HIL-7R-M21 clone). Fluorescence was measured with a LSRFortessa^TM^ flow cytometer and analyzed using FlowJo (software 10.9.0). Cell sorting was performed on a BD FACSAria™ III Cell Sorter.

### ELISA

The levels of IL-10 and total TGF-β1 were quantified in cell culture supernatants by specific sandwich ELISA (BioLegend and R&D systems, respectively), according to the manufacturer's guidelines.

### *In vivo* models

All animal experiments were performed in accordance with the European guidelines for the care and use of laboratory animals. All animal procedures were approved by the ≪ Ministère de l'Enseignement Supérieur et de la Recherche ≫ for germ-free mouse model: APAFIS#9232-2016122915218586 v6 and APAFIS#5110862024091713129725 v2 performed at Anaxem platform of the MICALIS Institute (INRAE, Jouy-en-Josas, France); and for the GvHD mouse model: APAFIS#9260-2017031514075189 v2 at Transcure (Archamps, France).

### Germ-free mouse model

The germ-free experiment was performed twice using 6–8 weeks-old male and female C3H/HeN germ-free mice. Microbial sterility of the mice was checked throughout the experiments. Briefly, fresh fecal samples and drinking water aliquots were collected and incubated in various culture conditions to assess potential growth of bacterial colonies. In addition, wet mounts of the fecal samples were observed under an optical microscope. Microbial sterility checking was performed before transferring the mice from the breeding unit to the experimental isolators, and 5 days after the transfer. This monitoring was continued in the germ-free group until the end of the experiment.

For the first experiment, germ-free mice were gavaged with 100 μL of the ready-to-use MaaT013 product (*n* = 8) or with PBS (*n* = 6) twice at D0 and D5 using flexible gavage needles. Fresh fecal pellets were collected from each mouse, distributed across two tubes, and stored at −80 °C pending analysis at D2, D4, D11, D14. At the termination day (D14), mice were deeply anesthetized with isoflurane and euthanized by cervical dislocation. The spleen and the colon were removed. Spleen was weighed and colon was fixed for histological analysis. All colon samples were stored in 70% ethanol at room temperature until analysis.

For the second experiment, germ-free mice were gavaged with 100 μL of the ready-to-use MaaT013 product (*n* = 8) or with PBS (*n* = 8) once at D0 using flexible gavage needles. C3H/HeNRj specific pathogen-free mice treated with PBS (*n* = 8) were used as controls. Fresh fecal pellets were collected from each mouse, distributed across two tubes, and stored at −80 °C pending analysis at D7, D14, D21 and D28. At the day of termination (D28), mice were deeply anesthetized with isoflurane and euthanized by cervical dislocation. The cecum was removed and weighed. The plasma and cecal content were collected and stored at −80 °C pending analysis. For both experiments, individual body weight was monitored throughout the experiment.

### Histological analysis

Flushed colons from GF study were fixed 24 h in 4% of paraformaldehyde. After fixation, samples were dehydrated in gradients of Ethanol (2 h in EtOH 80%, 2 h in EtOH 95%, 2 h in EtOH 100%), cleared in xylene (2 × 2h) and embedded in paraffin wax (3 × 2h). Five-micrometer thick sections were mounted on adhesive slides (Klinipath- KP-PRINTER ADHESIVES) and stained using a standard protocol of hematoxylin (C0303, DIAPATH, UGAP), eosin (C0353/U, Diapath) and saffron (F/SAFRAN, MM France) (HES) to study the morphology of tissues. Blue Alcian pH 2.5 staining (C0032, DIAPATH) with Nuclear Fast Red (C0482 DIAPATH) counterstained was performed to reveal Goblets cells. Colon tissues were visualized using a high-capacity digital slide scanner (Pannoramic Scan 150, 3DHISTECH Ltd.) and CaseViewer software (3DHISTECH Ltd.). The measure and analysis of the depth of crypts were performed blindly. U-shaped longitudinally cut crypts with open lumina along the crypt axis were selected and analyzed. Results report means from analyzing two colon sites of one slice for each mouse (*n* = 6 PBS treated germ-free mice and *n* = 8 MaaT013 treated mice) and 7 to 10 crypts per site (Wrzosek et al., 2013). The measure and analysis of the percentage of Goblet cells per crypt were performed blindly. U-shaped longitudinally cut crypts with open lumina were considered to count the total number of intestinal cells and Goblet cells per crypt. The percentage of Goblet cells was calculated by multiplying the Goblet cells number by 100 and then dividing it by the total number of intestinal cells per crypt. Results represent means from the analysis of two colon sites of one slice for each mouse (*n* = 6 germ-free mice and *n* = 8 MaaT013 treated mice) and 6–9 crypts per site (Wrzosek et al., 2013).

### GvHD mouse model

8 week-old female NOD/Shi-scid/IL-2Rγnull mice were treated with metronidazole (40 mg/kg) for 10 days in the drinking water (from D-14 to D-4), prior randomization and treatment initiation with MaaT013, to free ecological space in the gastrointestinal tract and facilitate the engraftment of MaaT013 microbes. Treatment with MaaT013 (*n* = 4) or with PBS (control group, *n* = 4) started on D-3 for 11 consecutive days and then once a week for 3 weeks. The treatment was given by oral gavage with an adequate oral gauge gavage tube (inoculation of 100 μL of the MaaT013 product suspension or PBS) into the stomach. At D0, mice were administered with human PBMCs via a single intraperitoneal injection (1 × 10^7^ PBMCs per mouse). Human PBMC expansion was monitored in all animals at D11 and D24 by flow cytometry analysis (Attune NxT, Life Technology) of 50 μL of blood using the following panel: hCD45 (Miltenyi Biotech), hCD4 (Miltenyi Biotech) hCD8 (Miltenyi Biotech). Clinical signs associated with GvHD were monitored until the end of the study (D46). On study days −4, 11, 17, 24 and 31, blood and feces were collected from all mice and stored at −80 °C until analysis. Throughout the experiment, mice were monitored daily for signs of distress. GvHD symptoms (including body weight and clinical signs) were recorded three times per week. Upon first signs of GvHD, the recording increased to five times per week. The score criteria were based on previous work (Nervi et al., 2007). Mice were sacrificed by cervical dislocation when they reached one of the following criteria: (i) > 20% of body weight loss or (ii) cumulative clinical GvHD score > 7. After death, the animals' last individual score was increased by 3 points (0.5 for each of the six criteria) and this adjusted score was kept unchanged until the end of the study. The reason for each death was reported. A survival curve reporting the death of all mice (spontaneous or ethical sacrifice) was performed. Plasma samples were collected at D-4, D11, D17 and D31 and stored at −80 °C. IL-10 was quantified using a LEGENDPLEX Multi-Analyte Flow Assay kit (BioLegend, 740929) according to manufacturer's instructions. Samples were acquired using an Attune NxT flow cytometer. Data were analyzed using the Legendplex software (BioLegend).

### Fecal microbiome analysis

#### DNA isolation, 16S rDNA and shotgun sequencing

Genomic DNA was extracted from fecal material using the NucleoSpin Soil kit (Macherey-Nagel), following the manufacturer's protocol. For samples from the first germ-free experiment and the GvHD mouse study, sequencing libraries targeting the V3–V4 region of the 16S rDNA were prepared using MyTaq HSMix (Bioline). Libraries were sequenced in paired-end mode (2 × 300 bp) on an Illumina MiSeq platform. For samples from the second germ-free experiment, shotgun metagenomic libraries were generated from each DNA extract and sequenced on an Illumina NovaSeq 2500 instrument (2 × 150 bp). Positive and negative controls were included throughout to verify reagent integrity and workflow performance. The workflow used for microbial ecosystem characterization aligns with previous methodological approaches applied to pooled microbiome therapeutics (Reygner et al., 2024).

#### 16 S rDNA bioinformatic analysis

Bioinformatic processing of 16S rDNA sequences was performed using standard methodology. After read merging with FLASH, sequences were quality-filtered using Trimmomatic (Bolger et al., 2014). Amplicons were clustered into Operational Taxonomic Units (OTUs) at 97% sequence identity, and taxonomic assignments were obtained using VSEARCH (Rognes et al., 2016) against the SILVA SSU database (release 128). To facilitate direct comparisons between samples, each dataset was rarefied to 60,000 sequences. Alpha- and beta-diversity metrics were computed in R (version 3.4.4) using the *vegan* and *phyloseq* packages. Microbial engraftment was defined as the number of OTUs originating from the MaaT013 product that were detected in fecal samples of treated germ-free mice, consistent with previous definitions applied to pooled microbiome ecosystem therapies (Reygner et al., 2024). In the GvHD model, engraftment required OTUs to be both product-derived and absent at baseline.

#### Shotgun bioinformatic analysis

Shotgun bioinformatic analysis was performed using standard methodology. In brief, after quality filtering using Trimmomatic (Bolger et al., 2014), host sequence decontamination was performed using Bowtie2 (Langmead and Salzberg, 2012). To ensure comparability, all samples were rarefied to the same sequencing depth. Taxonomic profiling was performed with Kraken2 (Wood et al., 2019) and the RefSeq genomic database (2020 release, http://www.ncbi.nlm.nih.gov/refseq/). α- and β-diversity indexes were calculated using R Statistical Software (http://www.R-project.org, version 3.6.3) using vegan and phyloseq packages. Functional profiling was then performed using the NGLess framework (Coelho et al., 2019) with the IGC gene catalog (Li et al., 2014) annotated with KEGG KO identifiers (Kanehisa, 2002). Finally, antibiotic resistance analyses were performed through gene mapping with Bowtie2 on MEGARes database (Lakin et al., 2017). Microbial engraftment was defined as the number of species originating from the MaaT013 product that were detected in the feces of MaaT013-treated germ-free mice.

### Metabolomic analysis on MaaT013 products and samples from Germ-free experiment

Metabolite analysis was carried out by MS-Omics (Vedbæk, Denmark) as previously described (Johnsen et al., 2017; Marfil-Sánchez et al., 2021; Belotserkovsky et al., 2025). The SCFA panel included acetic, formic, propanoic, 2-methylpropanoic, butanoic, 2-methylbutanoic, 3-methylbutanoic, pentanoic, 2-methylpentanoic, 3-methylpentanoic, 4-methylpentanoic, hexanoic, and heptanoic acids. The BA panel used quantify the following metabolites: Ursocholic acid, Tauro-alpha-Muricholic acid, Tauro-beta-Muricholic acid, omega-Muricholic acid, alpha-Muricholic acid, beta-Muricholic acid, Glycoursodeoxycholic acid, Tauroursodeoxycholic acid, Taurolithocholic acid sulfate, Taurohyodeoxycholic acid, Hyocholic acid, Glycocholic acid 3-Oxocholic acid, Ursodeoxycholic acid, Taurocholic acid, Hyodeoxycholic acid, Cholic acid, 12-Ketolithocholic acid, Glycochenodeoxycholic acid, Taurochenodeoxycholic acid, Glycodeoxycholic acid, Taurodeoxycholic acid, 3-Oxochenodeoxycholic acid, Chenodeoxycholic acid, Glycolithocholic acid, Taurolithocholic acid, Deoxycholic acid, Isolithocholic acid, Lithocholic acid. The last panel included: Succinic acid, Lactic acid, Fumaric acid, Alanine, Glycine, Valine, 2-Picolinic acid, 2-Oxoglutaric acid, Leucine, Isoleucine, Indole, Phosphoenolpyruvic acid, Threonine, Malic acid, Proline, 3-Methylindole, cis-Aconitic acid, Asparagine, 5-Aminovaleric acid, Aspartic acid, Citric acid, Serine, Isocitric acid, 2,3-Pyridinedicarboxylic acid, Glutamic acid, Methionine, 4-Hydroxyphenylacetic acid, Phenylalanine, 3-(4-hydroxyphenyl)propionic acid, 4-Aminobenzoic acid, Anthranilic acid, Indole-3-pyruvic acid/Indole-3-acetic acid, Glutamine, Ornithine, Indole-3-propionic acid, Lysine, Indole-3-butyric acid, Histidine, Tyrosine, Tryptamine, Indole-3-acrylic acid, Tryptophan, Cystine.

### Transcriptomic analysis

#### RNA extraction and library preparation

Transcriptomic analysis was carried out by Explicyte (Bordeaux, France). Total RNA was extracted from frozen colon samples using Nucleozol (Macherey Nagel) and a bead mill homogenizer (VWR), followed by purification with the NucleoSpin RNA kit (Macherey Nagel) according to the manufacturer's protocol. RNA integrity was assessed on the Bioanalyzer (Agilent Technologies), and concentration was quantified using Qubit (ThermoFisher). RNA-Seq libraries were prepared from poly(A)-enriched mRNA using oligo(dT) primers. And a Unique Dual Index (UDI) system. Library quality was verified by qPCR and capillary electrophoresis; only libraries meeting concentration and fragment size criteria were sequenced.

#### Sequencing and data processing

Sequencing was performed on an Illumina NovaSeqX+ platform in paired-end mode (2 × 100 bp), targeting ~20 million read pairs per sample (~4 Gb/sample). Raw data were converted to FASTQ and demultiplexed using bcl2fastq v2.20 (one mismatch allowed). Adapter trimming and quality filtering were done with Trimmomatic v0.36 (Bolger et al., 2014). Reads were aligned to the mouse reference genome (GRCm39) using STAR v2.5.2b, and gene-level counts were obtained with featureCounts (Subread v1.5.2), retaining uniquely mapped reads overlapping annotated exons. QC included assessment of FASTQ quality, mapping statistics, and Q30 scores (>70–80% bases recommended). Mapping rates to the reference genome were computed to ensure data robustness.

#### Analyzed parameters

Differential expression analysis was performed using the DESeq2 R package. The resulting *P* values were adjusted using Benjamini and Hochberg's approach for controlling the False Discovery Rate (FDR). Genes with an adjusted *P* value < 0.05 and an absolute value of the logFoldChange > 1 found by DESeq2 was assigned as differentially expressed. To decipher the mechanism(s) triggered by the groups, enrichment analysis was performed using the clusterProfiler R package and by interrogating two different databases: Gene Ontology (GO) which annotates genes to “biological processes”, “molecular functions” and “cellular components”.

#### Statistical analysis

For *in vitro* experiments, data were analyzed using one-way ANOVA or Kruskal-Wallis test followed by Dunn's multiple comparisons test. For germ-free *in vivo* experiments, histological and physiological parameters were analyzed using a Mann-Whitney test. For all tests, the level of significance was set at *p* < 0.05. Calculations were performed using GraphPad Prism software (version 9.5.1.733, La Jolla, CA, USA).

#### Statistical analyses of metabolomic and metagenomic data

##### Data preparation

Metabolomic data from fecal and plasma samples were log-transformed using log(x + 1) to stabilize variance and normalize distributions. Features were then filtered out using the nearZeroVar function from the R caret package (default parameters: frequency ratio > 95/5 = 19 and percent unique values < 10%) to remove metabolites with near-zero variance, ensuring sufficient variability for downstream multivariate analyses. For heatmap visualizations, log-transformed metabolite abundances were further Z-score normalized across samples.

Metagenomic data (genus-level taxonomic abundances and MaaT indexes including butycore, core microbiota, and engraftment metrics) were log-transformed using log(x + 1). Taxonomic features with zero variance or near-zero variance (using the same nearZeroVar criteria) were identified and excluded from the analysis.

The resulting filtered dataset retained only features with sufficient inter-sample variability to focus on only relevant ones regarding comparison with others.

##### Multivariate (PERMANOVA) analysis

To assess global differences in metabolomic profiles between groups, we performed PERMANOVA using the adonis2 function from the R (version 4.4.2) vegan package. Metabolite data (log-transformed and Z-scored) were arranged as samples × metabolites. A Euclidean distance matrix was computed, and group differences were tested with 999 permutations.

##### Bi-clustered heatmap analysis

Heatmaps were generated using the pheatmap R package. Metabolite abundances were generated to visualize differences between the Germ Free (GF) and MaaT013 groups. Prior to visualization, metabolite concentrations were standardized using Z-score transformation to center and scale each metabolite across samples. Only metabolites found to be significantly different between groups (FDR-adjusted Wilcoxon test, *p* < 0.05) were included in the heatmaps. For each heatmap, both metabolites (rows) and samples (columns) were simultaneously clustered (biclustering) using hierarchical clustering, allowing patterns of co-variation to be identified across both dimensions. This approach highlights group-specific metabolite signatures and sample similarities.

##### Correlation analysis

For each pair of metabolites (row) and metagenomic feature (column), a Spearman correlation was computed. The corresponding *p*-value for each correlation was also calculated. Multiple testing correction was not applied at this stage, but significance was visually encoded in the bi-clustered heatmaps. Only metabolites and metagenomic features with at least one significant correlation are shown in the filtered heatmaps.

## Results

### MaaT013-derived metabolites prevent inflammation-induced gut barrier alteration in an *in vitro* leaky gut model

The gut epithelial barrier maintains the balance between immune tolerance and activation (Rescigno, 2011). In GI-aGvHD, barrier disruption allows luminal contents to trigger excessive immune responses and systemic inflammation (Ji et al., 2024; Vincenzo et al., 2024). A healthy gut microbiota supports barrier integrity through microbial metabolites that reinforce tight junctions, stimulate mucin production, and exert local anti-inflammatory effects (Ghosh et al., 2020). To evaluate the impact of MaaT013 on intestinal barrier integrity and inflammation, we first analyzed the metabolomic profile of MaaT013 product, focusing on key microbial-derived metabolites associated with health benefits such as SCFAs and BAs. MaaT013 contained acetate (9.04 ± 1.35 mM), propionate (2.86 ± 0.50 mM), and butyrate (2.64 ± 0.48 mM) in a 60:20:20 ratio, consistent with human fecal profiles (Cummings et al., 1987). It also exhibited secondary BAs (deoxycholic, 194.2 ± 89.7 μM, and lithocholic acids, 130.1 ± 65.7 μM) and other metabolites such as glutamic acid, 0.331 ± 0.08 mM, alanine, 0.198 ± 0.06 mM, and lysine, 0.107 ± 0.04 mM (Figure 1A).

**Figure 1:**
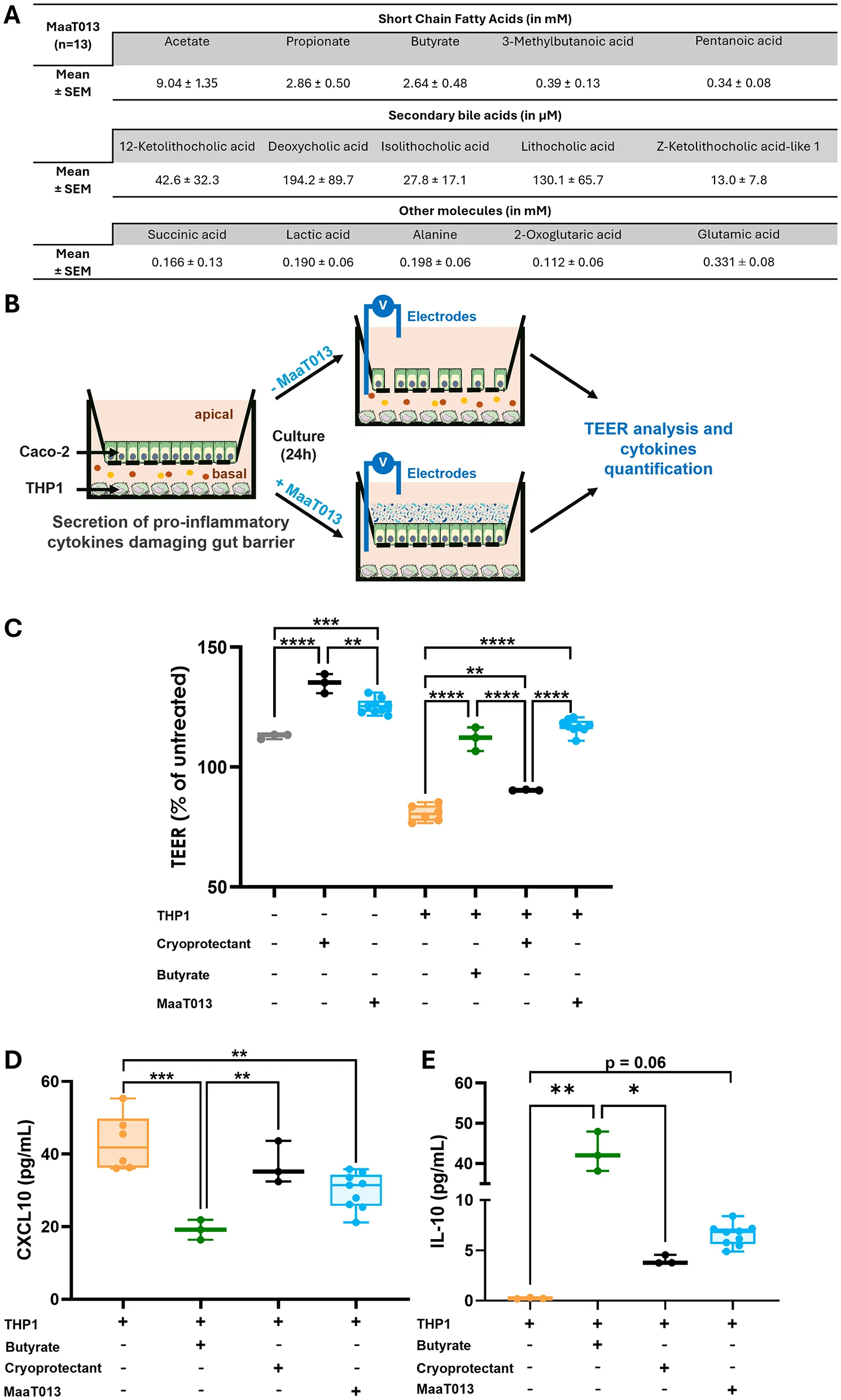
MaaT013-derived metabolites modulate cytokine responses and protect gut barrier integrity in an *in vitro* leaky gut model. **(A)** Metabolomic analysis of MaaT013 batches with the top 5 of the most abundant metabolites for SCFA, secondary bile acids and others. Data are presented as mean ± SEM of *n*=10 to 13 batches. **(B)** Leaky gut experimental design. **(C)** Healthy gut (Caco-2 cells culture) and leaky gut (Caco-2/THP1-Blue™ co-cultures) assays were performed in presence of MaaT013 supernatants (*n*=3 batches), or cryoprotectant, sodium butyrate and Caco-2 complete medium as controls. **(D)** Quantification of pro-inflammatory chemokine CXCL10 and **(E)** anti-inflammatory cytokine IL-10. Data are presented as mean ± SEM. A one-way ANOVA was performed followed by Tukey's multiple comparisons for statistical analysis. Adapted from Servier Medical Art (https://smart.servier.com), licensed under CC BY 4.0.

An established *in vitro* leaky gut model (Sapp et al., 2024) was used to mimic the gut epithelial barrier, with barrier integrity measured via transepithelial electrical resistance (TEER; Figure 1B). Briefly, Caco-2 cells were allowed to differentiate into mature enterocytes for 14 days in semi-permeable inserts. The apical compartment containing Caco-2 cells was then treated with MaaT013 supernatant (containing microbial-derived metabolites) or cryoprotectant as a control for 1 day before TEER analysis. Both cryoprotectant control and MaaT013 supernatants showed a significantly increased TEER compared to the untreated Caco-2 barrier (Figure 1C).

Next, the Caco-2-bearing inserts were placed on top of phorbol myristate acetate (PMA)-differentiated THP1 macrophages to mimic an inflammation-induced disruption of the gut barrier and to evaluate the potential of MaaT013 to prevent inflammation-induced gut barrier disruption. After 1 day of co-culture in presence or absence of MaaT013 supernatant or cryoprotectant as a control, TEER analysis was performed. We observed a statistically significant 30% decrease (*p* < 0.0001) in the TEER of Caco-2 cells due to the damage induced by PMA-activated THP1 macrophages (Figure 1C). While control cryoprotectant slightly increased the TEER values, MaaT013 supernatant and the butyrate positive control (12 mM) showed a significant protective effect, restoring TEER values to the level of untreated control. Further, MaaT013 supernatant showed a significantly greater effect on restoration of barrier than cryoprotectant alone (*p* < 0.0001, MaaT013 vs. cryoprotectant), comparable to the effect of butyrate (*p* = 0.1, MaaT013 vs. butyrate).

At the end of the co-culture, the basolateral medium was collected for quantification of CXCL10 and MCP-1 pro-inflammatory chemokines, IL-6 pleiotropic cytokine with both pro-inflammatory and pro-repair functions in the gut, and interleukin-10 (IL-10) anti-inflammatory cytokine. IL-6 and MCP-1 levels were unaffected by MaaT013 stimulation, with no significant difference compared to altered barrier control (Supplementary Figures 1A, B). A significant decrease of CXLC10 release was observed in response to MaaT013 supernatant and butyrate positive control compared to the altered gut barrier control (Figure 1D). A non-significant decrease of CXCL10 was also observed in response to cryoprotectant control. MaaT013 supernatant and butyrate positive control also significantly improved IL-10 release compared to both altered gut barrier and cryoprotectant controls (Figure 1E). In conclusion, MaaT013-derived metabolites were able to prevent inflammation-induced gut barrier disruption.

### MaaT013 promotes antigen-presenting cells (APC)-mediated Treg amplification and secretion of anti-inflammatory mediators

Innate immune cells sense commensal and pathogenic bacteria via pattern recognition receptors (PRRs), including toll-like receptors (TLRs) and nucleotide-binding and oligomerization domain (NOD) receptors expressed on gut epithelial and immune cells (Armant and Fenton, 2002). These receptors mediate microbial recognition and maintain mucosal immune homeostasis. To assess whether MaaT013 activates innate immune signaling as part of its biological activity, HEK-Blue reporter cells specific for TLR2, TLR4, TLR5, NOD1, and NOD2 were exposed to MaaT013 supernatant for 24h. Supernatants analyzed by QUANTI-Blue™ colorimetric assay showed consistent receptor activation compared with untreated and cryoprotectant controls (Supplementary Figure 2A).

Next, several *in vitro* assays were performed to evaluate the impact of MaaT013 on DC function, Treg amplification and secretion of anti-inflammatory mediators (Figure 2A). First, CD14^+^ monocytes were sorted from peripheral blood mononuclear cells (PBMCs) of healthy donors and differentiated into monocyte-derived dendritic cells (moDCs) by a 5- to 6-day culture in a medium containing rhGM-CSF and rhIL-4. Immature moDCs were then exposed to heat-inactivated MaaT013 bacteria (ratio 1 DC: 25 bacteria) or control cryoprotectant for 48 h before flow cytometry analysis. MaaT013 showed a tendency to up-regulate the expression of molecules associated with DC maturation (CD83, *p* = 0.016; Figure 2B), antigen presentation (HLA class II, *p* = 0.016; Figure 2B), and co-stimulation (CD80, *p* = 0.071; CD86, *p* = 0.60; CD40, *p* = 0.12; Figure 2C), as well as Treg polarization molecules (CD39, *p* = 0.065; PD-L1, *p* = 0.0004; Figure 2D). Remarkably, heat-inactivated MaaT013 bacteria significantly induced the production of high levels of IL-10 by moDCs (Figure 2D).

**Figure 2:**
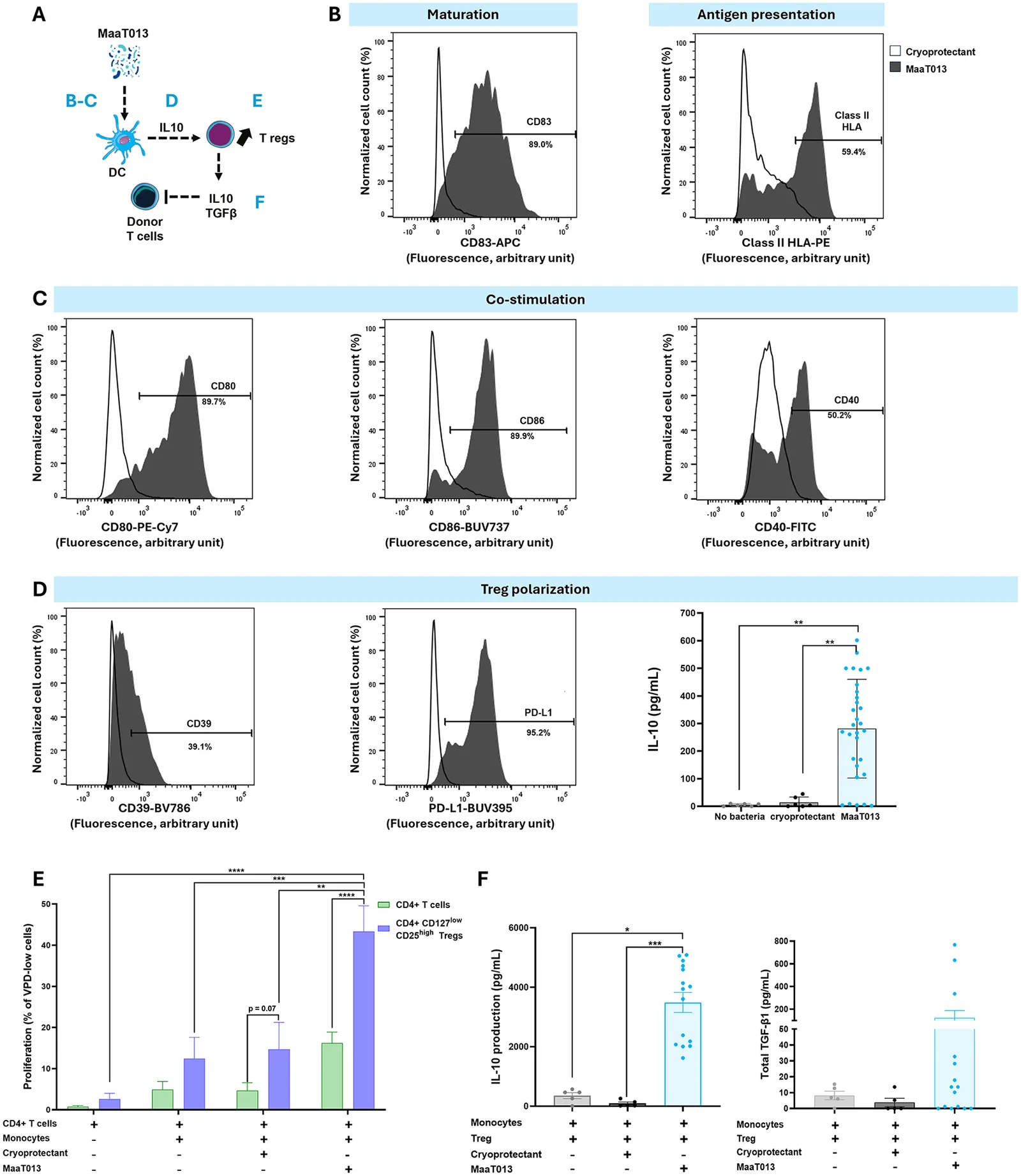
MaaT013 stimulates dendritic cell maturation, expression of Treg polarization molecules and Tregs amplification *in vitro*. **(A)** Experimental design. **(B, C)** Immature monocyte-derived DCs conditioned with MaaT013 were analyzed by flow cytometry (CD80, CD83, CD86, CD40, class II HLA). **(D)** Expression of Treg polarization molecules was analyzed by flow cytometry (CD39, PD-L1) and secretion of anti-inflammatory cytokine IL-10 by MaaT013-primed moDCs was measured by ELISA. **(E)** MaaT013-exposed monocytes cultured in presence of autologous CD4+ cells (3 MaaT013 batches tested in PBMCs from 5 healthy donors). VPD analysis was performed to quantify CD4+ T cells and CD3+ CD4+ CD25+ CD127low Tregs proliferation by flow cytometry. **(F)** Secretion of anti-inflammatory cytokines IL-10 and TGF-β by Tregs activated by MaaT013-primed monocytes. All data are presented as mean ± SEM. 5 MaaT013 batches tested in 6 healthy donors in B-D and 3 MaaT013 batches tested in PBMCs from 5 healthy donors in E-F. Kruskal-Wallis test was performed followed by Dunn's multiple comparison test for all experiments except for Treg proliferation, a two-way ANOVA was performed followed by a Tukey's multiple comparison test. *P* < 0.05 *; *p* < 0.005 **; *p* < 0.0005 ***; *p* < 0.0001 ****. Adapted from Servier Medical Art (https://smart.servier.com), licensed under CC BY 4.0.

To evaluate the potential of MaaT013 to modulate Tregs activation, CD4^+^ CD25^high^ CD127^low^ Tregs and single positive CD4^+^ T cells were sorted from healthy PBMCs and stained with Violet Proliferation Dye 450 (VPD) before being co-cultured with autologous CD14^+^ monocytes previously loaded with heat-inactivated MaaT013 bacteria overnight (ratio 1 monocyte: 5 bacteria). The gating strategy for CD4^+^ CD25^high^ CD127^low^ Treg selection is presented in Supplementary Figure 2B. Conventional CD4^+^ CD25^high^ CD127^low^ Tregs were selected as the optimal live-cell population compatible with downstream proliferation assays. This phenotype is a well-validated surrogate for Foxp3^+^ Tregs, providing robust enrichment for Foxp3^+^ suppressive Tregs without requiring fixation/permeabilization necessary for intracellular FOXP3 staining which would preclude functional readouts (Liu et al., 2006). After 5 days, the proliferation of single positive CD4+ T cells and CD4^+^ CD25^high^ CD127^low^ Tregs was analyzed through VPD dilution by flow cytometry. While control cryoprotectant did not influence T cell proliferation, MaaT013 bacteria-loaded monocytes were able to significantly induce both single positive CD4^+^ T cell and CD4^+^ CD25^high^ CD127^low^ Treg proliferation (Figure 2E). Strikingly, the percentage of proliferating cells reached 43% for CD4^+^ CD25^high^ CD127^low^ Tregs compared to 16% for total single positive CD4^+^ T cells. This amplification of Tregs was associated with a significant release of both TGF-β1 and IL-10 anti-inflammatory cytokines (Figure 2F). Altogether, these results demonstrate that MaaT013 may modulate immune response by promoting dendritic cell maturation and the expression of Treg polarizing molecules, resulting in Treg expansion and increased secretion of anti-inflammatory mediators.

### MaaT013 safely engrafts in germ-free mice, restoring healthy gut physiology and immune homeostasis

Germ-free mice are considered as the model of reference to study fecal microbiotherapies in animals as they are completely devoid of microorganisms (Kennedy et al., 2018). Germ-free mice were therefore used to investigate the engraftment of bacteria present in MaaT013, which is considered as the first necessary step to achieve its biological activity. Two experiments were performed: the first experiment lasted 14 days and included histological endpoints, the second experiment lasted 28 days and included fecal microbiome analysis, metabolomic and transcriptomic analyses (Figure 3A). For fecal microbiome and metabolomic analyses, SPF mice were used as a positive control for healthy endogen microbiota.

**Figure 3:**
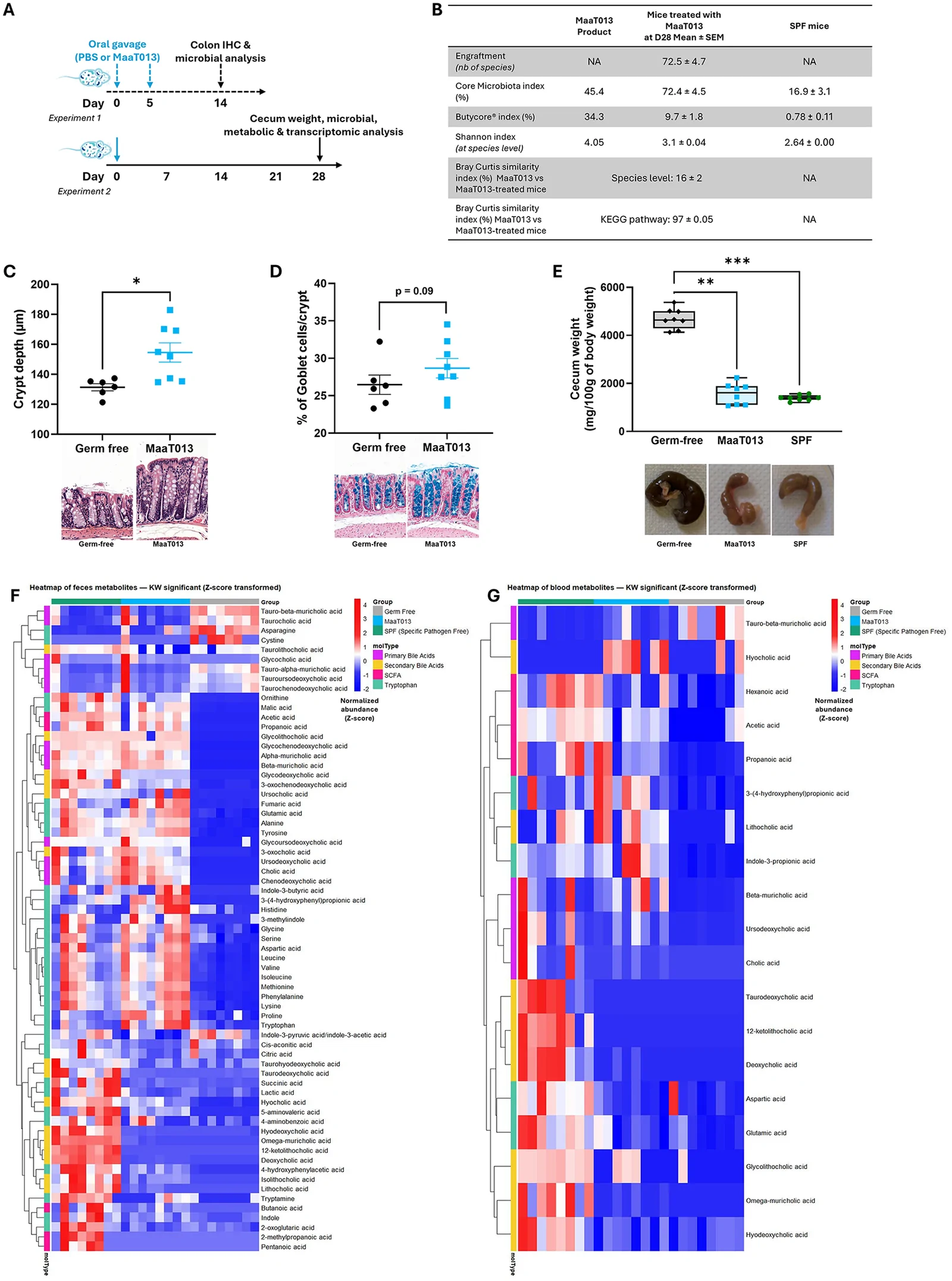
MaaT013 safely engrafts in germ-free mice, restoring healthy gut physiology, gut microbial functions and production of key microbial-derived metabolites. **(A)** Experimental design. 6-8 weeks old male and female C3H/HeN germ-free mice were used. In experiment 1 (dash arrow), mice were dosed twice via oral gavage with 100 μL of MaaT013 (10^8^ viable bacteria) (8 mice) or sterile water as control at day 0 and day 5. In experiment 2, mice were dosed once via oral gavage with 100 μL of MaaT013 (10^8^ viable bacteria) (8 mice) or sterile water as control at day 0. SPF mice treated with PBS (n=8) were used as controls. **(B)** Shotgun sequencing was used to determine key microbial characteristics of MaaT013 product, fecal microbiota from MaaT013-treated mice and SPF mice at D28. The Butycore^®^ (MaaT Pharma proprietary index) is the sum of the relative abundances of a set of different genera (*Blautia, Faecalibacterium, Alistipes, Eubacterium, Bifidobacterium, Ruminococcus, Clostridium, Coprococcus, Odoribacter, Roseburia, Holdemanella, Anaerostipes, Oscillibacter, Subdoligranulum, Butyrivibrio*) known to produce short-chain fatty acids (mainly butyrate) with anti-inflammatory properties in human. The Core microbiota (MaaT Pharma proprietary index) is the sum of the relative abundances of a set of 11 genera (*Alistipes, Bacteroides, Blautia, Coprococcus, Dorea, Faecalibacterium, Lachnospira, Parabacteroides, Roseburia, Ruminococcus, Subdoligranulum*) found in most healthy human individuals. **(C)** Colonic crypt depth in MaaT013-treated and germ-free mice. Representative examples of hematoxylin-eosin stained colon tissues from germ-free and MaaT013-treated mice are provided. Scale bar indicates 20 μm. **(D)** Percentage of Goblet cells per colonic crypt in MaaT013-treated and germ-free mice. Representative examples of alcian blue stained colon tissues of germ-free and MaaT013-treated mice are provided. Scale bar indicates 20 μm. **(E)** Cecum weight at the time of necropsy (D28) expressed as mg/100 mg of body weight. Representative images of the cecum from germ-free, MaaT013-treated mice and SPF mice are provided. Data are presented as mean ± SEM. Mann-Whitney test was performed. *P* < 0.05 *; *p* < 0.005 **; *p* < 0.0005 ***; *p* < 0.0001 ****. **(F)** Hierarchical bi-clustering analysis revealing differential metabolite levels in the feces of germ-free, MaaT013-treated mice and SPF group. **(G)** Hierarchical bi-clustering analysis revealing differential metabolite levels in the plasma of germ-free, MaaT013-treated mice and SPF group.

The taxonomic and functional profiles of mice were evaluated using shotgun sequencing of feces at day 28. Substantial microbial engraftment (72.5 species on average) was observed in MaaT013 treated mice. As expected, the microbial features of MaaT013 product were transferred to MaaT013-treated mice with some variations in microbial relative abundances highlighted by Core Microbiota index, Butycore^®^ index and Shannon index (Figure 3B and Supplementary Figure 3) compared to MaaT013 product and to SPF positive control group. At functional level, MaaT013-treated mice displayed a KEGG pathway profile remarkably similar to that of corresponding MaaT013 product (97% functional similarity; Figure 3B). These results illustrate the host-specific taxonomic reshaping of the human-derived microbial community upon adaptation to the murine intestinal environment, resulting in a microbiota profile clearly distinct from the endogenous SPF mouse microbiota and characterized by an increased diversity and enrichment of key functional bacterial groups (Beresford-Jones et al., 2022). At necropsy, colon and cecum were collected to evaluate morphological and metabolic changes following MaaT013 engraftment. A significant increase in the depth of intestinal crypts was observed for MaaT013-treated mice compared to germ-free control groups (154.5 ± 6.4 μm for MaaT013 and 131.3 ± 2.4 μm for control mice, *p* = 0.0023; Figure 3C), suggesting an improvement of gut physiology (Vangay et al., 2023). Mice treated with MaaT013 also showed a trend toward an increase in the percentage of goblet cells compared to the germ-free control group (28.7 ± 1.3% for MaaT013 and 26.5 ± 1.3% for control mice, *p* = 0.09; Figure 3D). In mice, the cecum is the main site of metabolic activity. Because of the absence of microorganisms, the cecum of germ-free mice can be up to five times larger than that of conventional mice due to undegraded mucopolysaccharides attracting water and leading to intestinal atony (Basic and Bleich, 2019). The cecal content of mice was weighed to follow microbial metabolic activity. After MaaT013 transfer, mice displayed a significant reduction in cecum weight compared to untreated germ-free mice (1,559 ± 156.6 mg/100 g of body weight for MaaT013 and 4,695 ± 149.1 mg/100 g of body weight for control mice, *p* ≤ 0.0001) reaching values comparable to those observed in SPF mice (1,393 ± 42.05 mg/100 g of body weight; Figure 3E). These results are in line with the literature (Kennedy et al., 2018) and suggest that microbes from MaaT013 successfully engrafted and remained metabolically active, thereby may improve food digestion and restore the cecum toward a healthy state, as reflected by decreased cecal content.

To characterize the metabolic changes induced by MaaT013 engraftment, SCFAs, BAs and tryptophan-derived metabolites were quantified in the feces and plasma of mice. MaaT013 administration markedly altered the fecal metabolome, with increased levels for 84.9% of the detected metabolites (45 out of 53) compared with control germ-free animals (Supplementary Figure 4A). Among these 45 metabolites, the three major SCFAs (butyrate, propionate, and acetate) accounted for 6.7% (3/45), bile acids for 40% (18/45), and tryptophan-derived metabolites for 53.3% (24/45; Figure 3F). Consistently, fecal metabolomic profile clearly distinguished GF, SPF, and MaaT013-treated mice. SPF mice were enriched in microbiota-derived metabolites, including SCFAs, secondary bile acids, and indole derivatives, whereas these compounds were strongly depleted in GF animals. Primary conjugated bile acids and amino acids accumulated in GF mice, reflecting absence of microbial biotransformation and substrate utilization (Masse and Lu, 2023; Zhang et al., 2025). MaaT013 administration induced a broad shift of the metabolomic profile toward the SPF state, characterized by increased levels of SCFAs, secondary BAs, and indole derivatives. This pattern is consistent with the restoration of microbial fermentative activity and cross-feeding networks, indicating recovery of coordinated, ecosystem-level functions rather than isolated pathways (Masse and Lu, 2023). In plasma, MaaT013 induced more limited changes, with only 8 metabolites increased compared to GF controls, predominantly BAs (62.5%, 5/8), followed by tryptophan-derived metabolites (25%, 2/8) and SCFAs (12.5%, 1/8; Figure 3G). Plasma metabolomic profiles also discriminated GF, SPF and MaaT013-treated mice, albeit with lower amplitude than in feces. SPF mice were enriched in circulating secondary BAs and indoles derivatives, whereas these metabolites were reduced in GF group. MaaT013 administration partially restored these systemic metabolites, indicating recovery of microbiota-dependent functions reaching the circulation, while SCFAs showed limited changes consistent with rapid host utilization (Li et al., 2021, 2022a; Fiorucci et al., 2026). Overall, these results suggest that MaaT013 engraftment reshapes the gut metabolome (and to a lower extent the systemic metabolome) toward increased production of SCFAs, bile acids—in particular secondary bile acids -, and tryptophan-derived metabolites associated with improved intestinal barrier function, anti-inflammatory activity, and reduced GvHD severity. Despite this global shift, both fecal and plasma metabolomic profiles of MaaT013-treated mice remained distinct from that of SPF mice, suggesting incomplete functional restoration. This partial shift toward the SPF state may reflect the limited adaptation of a human-derived microbiota to the murine host environment and its associated ecological constraints (Wong et al., 2025).

Correlations were observed between the abundance of bacterial genera in the feces of MaaT013 treated mice and the abundance of specific fecal metabolites. The relative abundance of a group of 13 bacterial genera (*Clostridium, Ruthenibacterium, Dysosmobacter, Eisenbergiella, Flavonifractor, Fusicatenibacter, Ruminococcus, Alistipes, Medierraneibacter, Odoribacter, Faecalitalea, Phascolarctobacterium*) was positively correlated to the abundance of SCFAs (acetic acid and propanoic acid) and secondary BAs (hyodeoxycholic acid, deoxycholic acid, hyocholic acid). The relative abundance of a second group of 5 genera (*Phocaeicola, Coprobacter, Parasutterella, Barnesiella* and *Hungatella*) was positively correlated with the abundance of the tryptophan-derived metabolites indole-3-butyrate and indole-3-propionate and secondary BAs. *Parabacteroides* was positively correlated with tryptophan-derived metabolites (indole-3-acrylate and indole-3-pyruvate/indole-3-acetate; Supplementary Figure 4B). Similarly in plasma, correlations were observed for two bacterial groups positively correlated with either secondary BAs and tryptophan-derived metabolites or SCFAs (Supplementary Figure 4C). These results highlight the importance of bacterial diversity for the establishment of a complex metabolic network.

Next, to assess host GI responses to MaaT013 microbial engraftment, transcriptomic analysis of colon tissue from MaaT013-treated mice was performed in experiment 2. MaaT013 administration induced a significant increase in the expression of 195 genes in the colon of mice (> 1-fold, *p* < 0.05; Figure 4A). MaaT013 treatment strongly upregulated immunoglobulin genes (Figure 4B), particularly *Igha* and *Jchain*, while increasing immunoglobulin diversity through elevated expression of multiple variable chains (*Ighv, Igkv*). Gene ontology analysis highlighted an increase in biological processes associated with innate and adaptative immune activation such as *BCR/CD22* pathway, which is involved in B cells activation and antibody production, and *Fc*γ*R/Fc*ε*RI*, which is implicated in the recruitment and activation of effector cells such as mast cells or macrophages, or the restoration of innate defense with complement and phagocytosis (Figure 4C). The impact of MaaT013 on colonic biology is further evidenced by the upregulation of genes involved in GI homeostasis and immune regulation, including *Igha, Jchain, Mzb1*, and *Ccr10*, which indicate enhanced IgA production and plasmocyte homing, thereby supporting the reconstitution of humoral defenses at mucosal surfaces (Figure 4D). Increased levels of *Vill* and *Btnl1* reflects improved epithelial integrity and strengthened γδ T cell – epithelial interactions. Upregulation of *Nfil3, Tigit, Arg2*, and *Areg* is consistent with a shift toward regulatory and tissue-repair programs that help limit excessive inflammation. The induction of *Cyp2c55* suggests metabolic adaptation involving bile acid metabolism and PPARα-associated signaling pathways. Finally, increased *Bhlha15* levels suggest an amplification of Paneth cells. Paneth cells exert a protective effect against lower GI aGvHD by safeguarding the intestinal barrier through secretion of antimicrobial peptides that preserve commensal bacteria while inhibiting pathogens.

**Figure 4:**
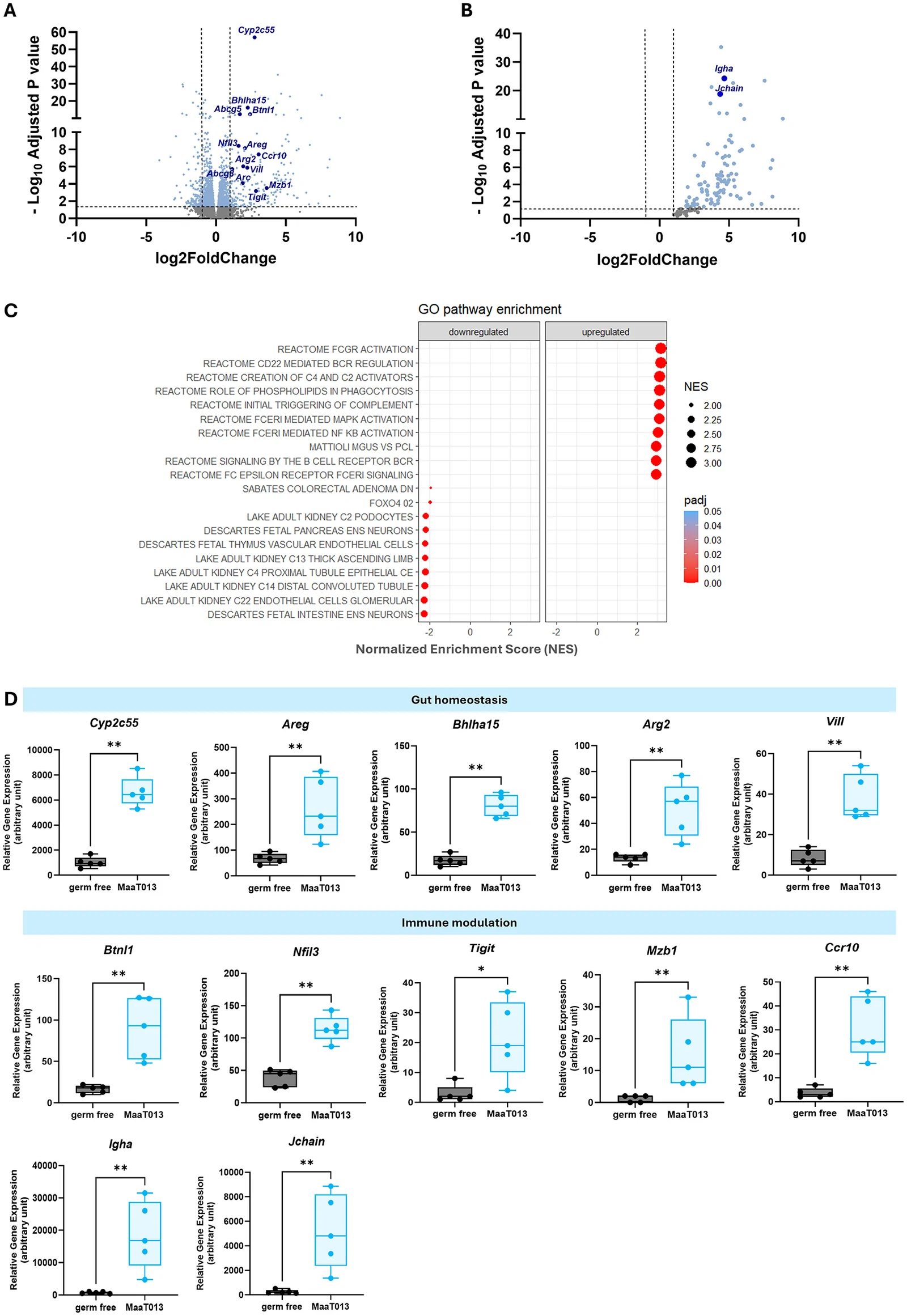
Transcriptomic profiling of colon tissue reveals that MaaT013 restores healthy gut homeostasis and control inflammation in germ-free mice. **(A)** Volcano plot analysis showing differentially expressed genes in the colon of germ-free mice 28 days after MaaT013 administration. **(B)** Volcano plot analysis showing differentially expressed immunoglobulin genes induced by MaaT013 administration in the colon of germ-free mice. **(C)** Gene ontology (GO) pathway enrichment analysis. **(D)** Bar plot analysis of genes involved in intestinal homeostasis and immune regulation. Mann-Whitney test was performed. *P* < 0.05 *; *p* < 0.005 **.

Collectively, these results demonstrate the ability of MaaT013-associated beneficial microbes to safely and durably engraft the GI tract of germ-free mice, and reveal associations with the production of key microbial-derived metabolites implicated in the regulation of GI physiology and immunity. These observations should be interpreted as correlative and hypothesis-generating, and further studies will be required to establish causal relationships and functional mechanisms.

### MaaT013 modulates the temporal progression of GvHD symptoms in SPF NCG mice humanized with human PBMCs

To explore the potential effects of MaaT013 in GvHD, the severely immunodeficient NOD/Shi-scid/IL-2Rγnull (NCG) mouse model was used. These SPF mice are characterized by severe defects in T-, B-, and natural killer cells, as well as poorly functioning macrophages and dendritic cells, allowing the expansion of human peripheral blood mononuclear cells (PBMCs). NCG mice humanized with human PBMCs often develop severe GvHD and have been used to study this disease (Hess et al., 2021). Briefly, mice were treated with the antibiotic Metronidazole for 10 days to prepare the ecological niche and allow MaaT013 engraftment. Mice were then treated with either MaaT013 or PBS as a control for 11 consecutive days (from D-3 to D7) followed by once a week for 3 weeks. At D0, mice received human PBMCs via a single intraperitoneal injection (Figure 5A). The evolution of 6 clinical signs (body weight loss, posture, activity, fur texture, skin integrity, paleness) was used to calculate GvHD scores (Nervi et al., 2007). In control mice, GvHD score gradually increased from D11 to D31. However, in MaaT013-treated mice, the appearance of GvHD symptoms appeared delayed until D27 (Figure 5B). In MaaT013-treated mice, GvHD scores tended to be lower than in control mice at D25 and D27, although the differences did not reach statistical significance (*p* = 0.0571 and *p* = 0.0857, respectively). From D27 onward, the GvHD score of MaaT013-treated mice increased rapidly to reach the same score as control mice at D31. No significant difference in survival between groups was observed.

**Figure 5:**
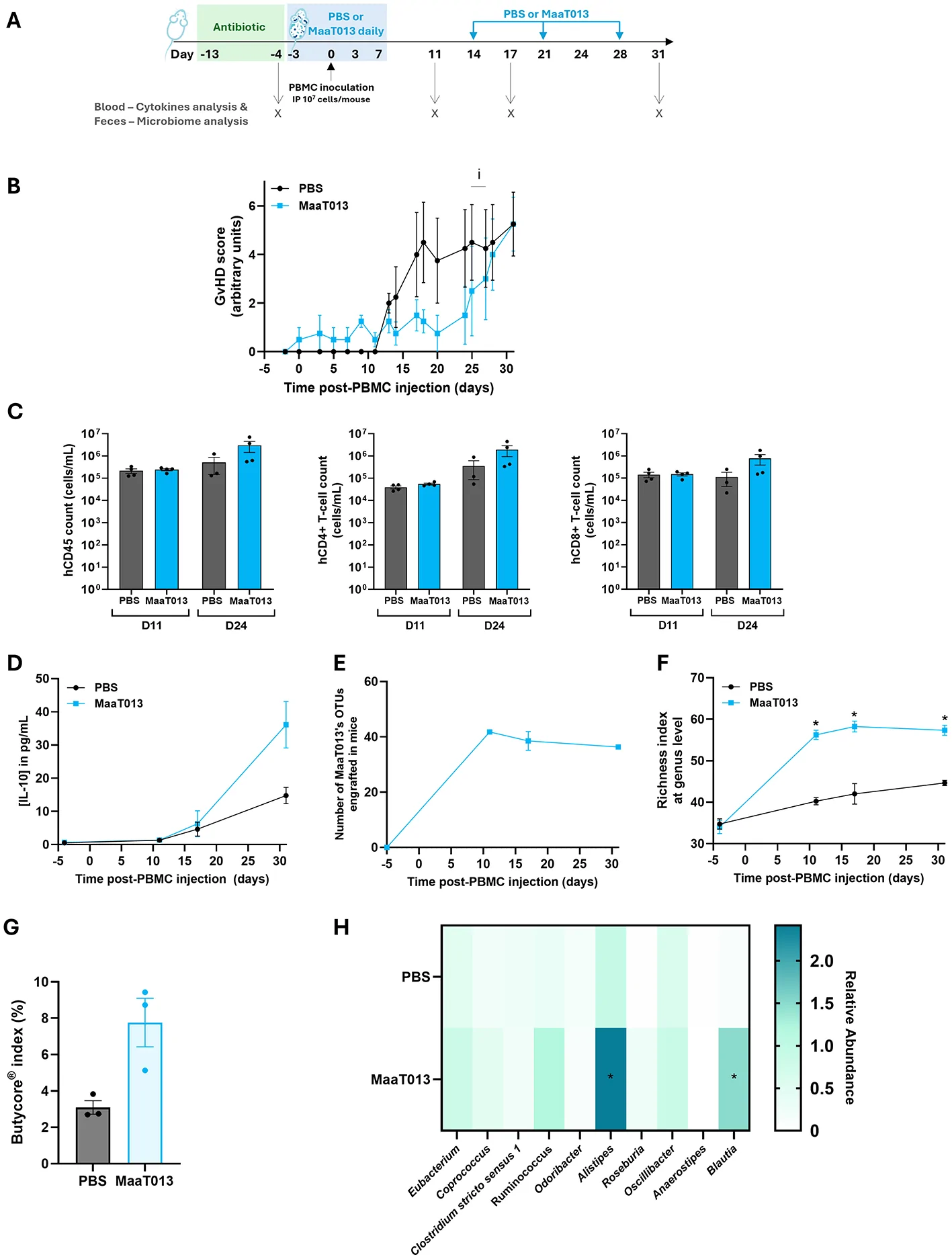
MaaT013 modulates the temporal progression of GvHD symptoms in SPF NCG mice humanized with human PBMCs. Experimental design. 8 weeks old severely immunodeficient female specific-pathogen-free NOD/Shi-scid/IL-2Rγnull (NCG) mice humanized with human PBMCs were used as a xenogeneic model of GvHD (4 mice per group). **(B)** The sum of six individual parameters (i.e. body weight loss, posture, skin integrity, fur texture, activity and paleness) was used to score GvHD progression. GvHD score between PBS and MaaT013-treated mice at D25 (*p* = 0.0571) and D27 (*p* = 0.0857). **(C)** Human PBMCs expansion in humanized SPF NCG mice treated with MaaT013 or PBS was analyzed by flow cytometry (hCD45, hCD4, hCD8). **(D)** IL-10 quantification in the plasma of mice. **(E)** MaaT013 engraftment in mice defined as the number of MaaT013 OTUs absent at baseline and detected in mice after treatment. **(F)** Microbial richness index at genus level. **(G)** Butycore^®^ index at D31. **(H)** Heatmap depicting relative abundance of beneficial bacteria from Butycore^®^ genera at D31. Data are presented as mean ± SEM. Mann-Whitney test was performed. *p* < 0.05 *.

Flow cytometry analysis was performed on blood samples collected at D11 and D24 to monitor the amplification of human CD45^+^, CD4^+^ and CD8^+^ T cells in mice. Overall, human CD45^+^, CD4^+^ and CD8^+^ T lymphocytes increased from D11 to D24, coinciding with GvHD onset in both groups. No significant difference was observed between PBS- and MaaT013-treated mice, suggesting that MaaT013 did not directly affect human T cell expansion (Figure 5C). Increased blood levels of anti-inflammatory cytokine IL-10 were observed in both groups in a time-dependent manner, with a trend toward higher levels in MaaT013-treated mice at Day 31 (Figure 5D).

To study changes in gut microbiota after MaaT013 administration, fecal samples were collected (D-4, D11, D17, D31) and analyzed using 16S rDNA sequencing. Microbial engraftment was measured as the number of OTUs present in MaaT013 and not present at baseline which were found in the feces of MaaT013-treated mice. A relatively stable microbial engraftment of MaaT013 was observed during the study (42 OTUs at Day 11, 39 OTUs at Day 17 and 36 OTUs at Day 31; Figure 5E). The taxonomic profile of MaaT013-treated mice closely mirrored that of MaaT013 product, as opposed to control PBS-treated mice (Supplementary Figure 5). MaaT013 administration increased gut microbial richness at the genus level compared to control mice, and this effect was maintained over time (Figure 5F). An increase in the fecal Butycore^®^ index (number of selected butyrate-producing genera) was observed in MaaT013-treated mice, rising from 3 in the control group to 8 at D31 (Figure 5G). I particular, MaaT013-treated mice showed significantly increased levels of *Alistipes* and *Blautia* (Figure 5H), two beneficial genera previously associated with improved intestinal homeostasis and anti-inflammatory profiles, and reported to correlate with reduced GvHD severity (Jenq et al., 2015; Malard et al., 2023b). These results show that MaaT013 engrafts in this model and is associated with changes in microbial composition and a transient delay in GvHD symptom onset. However, these observations remain correlative and do not establish a causal role of specific microbial taxa in modulating disease progression.

## Discussion

In this study, *in vitro* and *in vivo* data on the pooled fecal microbiotherapy MaaT013 using multiple models provides exploratory insights into biological processes that may contribute to clinical effects. Specifically, MaaT013 is shown to exhibit high microbial diversity and overall taxonomic consistency, while preserving key gut microbial functions comparable to those of corresponding single-donor feces (Supplementary Figure 6). MaaT013 also showed high batch-to-batch consistency in key biological features, including consistent microbial composition (Supplementary Figure 6), metabolite profiles (Figure 1A, Supplementary Figure 6F), gut barrier repair capacity (Figures 1B–E), and immune modulatory effects leading to DC-mediated Treg amplification (Figure 2, Supplementary Figure 7).

Complementary approaches were used to investigate putative complex host–microbiome interactions underlying MaaT013 activity, which could not be fully recapitulated in a single model. *In vitro* systems relied either on MaaT013-derived supernatants, which capture the effects of a broad range of microbial metabolites, or on heat-inactivated bacteria, which allow assessment of microbial uptake, processing, and antigen presentation independently of active bacterial metabolism. This design was driven both by experimental constraints, since the strict anaerobic requirements of MaaT013 bacteria are incompatible with the aerobic conditions required for cell-based assays, and by the need to dissect distinct mechanistic layers (Norton et al., 2021; Fofanova et al., 2024; Moore et al., 2025). In contrast, *in vivo* models using live bacteria captured integrated ecosystem-level processes, including colonization, metabolism, and host–microbiome interactions.

MaaT013 was associated with increased microbial diversity in both germ-free and SPF mice models, by engrafting key taxa: primarily *Bacteroides* and Lachnospiraceae, both major SCFA producers. As anticipated, however, not all MaaT013-derived bacteria colonized the murine colon, reflecting physiological disparities between human and mouse intestinal ecosystems (Arrieta et al., 2016; Clavel et al., 2016). In germ-free and SPF mice, MaaT013 administration significantly increased levels of *Blautia* and *Alistipes*, two genera associated with improved aGvHD outcome in patients (Jenq et al., 2015; Malard et al., 2023b). Non-significant trends toward increased abundance of other beneficial taxa, including *Eubacterium, Coprococcus, Ruminococcus, Roseburia*, and *Oscillibacter*, were also observed in SPF NCG mice humanized with human PBMCs to model GvHD. This shift in microbial composition was associated with a temporal trend toward lower GvHD severity at intermediate time points. It should be noted that although mouse models are widely used, their predictivity for evaluating human fecal microbiotherapies is limited by substantial microbiological, physiological, and immunological differences between species.

In aGvHD, gut dysbiosis is associated with a disruption of microbial metabolism. In particular, reduced fecal concentrations of the SCFAs butyrate and propionate have been reported in both patients and GvHD mouse models (Mathewson et al., 2016; Fujiwara et al., 2018; Romick-Rosendale et al., 2018; Markey et al., 2020; Payen et al., 2020). In experimental models, supplementation with butyrate, butyrate-producing bacteria or propionate mitigated aGvHD severity by protecting intestinal epithelial cells (IECs) and restoring gut barrier integrity (Mathewson et al., 2016; Fujiwara et al., 2018). The loss of key SCFA producers, including Lachnospiraceae, Ruminococcaceae, Erysipelotrichaceae (butyrate producers), and *Blautia* (a propionate producer), supports a mechanistic link between intestinal dysbiosis and SCFA depletion in aGvHD. SCFAs are derived from fermentation of carbohydrates by anaerobic commensal bacteria (Fusco et al., 2023) and support key physiological processes, including epithelial integrity, histone acetylation in IECs, and Treg induction. SCFAs serve as an energy source for IECs (Guilloteau et al., 2010), stimulate antimicrobial peptide secretion by Paneth cells (Takakuwa et al., 2019), and enhance mucus production by goblet cells (Wrzosek et al., 2013), thereby preventing pathogen translocation (Fukuda et al., 2011). SCFAs also promote immune tolerance by inhibiting histone deacetylases in immune cells, favoring tolerogenic epigenetic programming (Kaisar et al., 2017; Zhang et al., 2024), and driving Treg differentiation and expansion (Furusawa et al., 2013; Smith et al., 2013), which induces anti-inflammatory cytokines like IL-10 to counter excessive inflammation. In this study, MaaT013-treated germ-free mice exhibited increased fecal butyrate, acetate, and propionate levels, alongside increased blood propionate. Fecal propionate positively correlated with *Fusicatenibacter*, a Firmicutes genus associated with IL-10 induction (Yu et al., 2022) and improved FMT outcomes in steroid-refractory cGvHD (Golob et al., 2017; Yang et al., 2023). Acetate levels correlated with *Paraprevotella*, which supports epithelial protection and prevents IgA degradation (Li et al., 2022b), and with *Faecalitalea, Odoribacter*, and *Mediterraneibacter*, which are all SCFA producers associated with mucosal tolerance and IL-10 secretion (Togo et al., 2018; Ma et al., 2020; Lima et al., 2022; Xie et al., 2022; Bosch et al., 2023).

Other key metabolites, such as tryptophan-derived indoles (e.g., indole propionate) and secondary bile acids—both critical for barrier function and immune regulation—are depleted during GvHD onset (Michonneau et al., 2019). SCFAs, indoles, and secondary BAs were detected in MaaT013 products and were substantially increased in germ-free mice following MaaT013 administration. This restoration of microbial metabolic activity was accompanied by reduced cecal content, increased colonic crypt depth, and higher goblet cell numbers in MaaT013-treated mice, consistent with epithelial remodeling and features associated with mucosal recovery. MaaT013 supernatants, comprising complex mixtures of microbial metabolites, were also associated with protection against inflammation-induced intestinal barrier alteration in an *in vitro* leaky gut model challenged by activated macrophages. Together, these findings support a link between MaaT013-associated microbial metabolic activity and markers of improved barrier integrity, a key feature of GI-aGvHD pathology. However, these observations remain correlative, and further mechanistic studies will be required to establish the causal contribution of specific metabolites or microbial components to the effects observed.

In aGvHD patients, the GI tract serves as a primary target for alloreactive donor T cells, with GI involvement portending poor prognosis due to reduced therapeutic responsiveness and amplification of systemic inflammation (Hill and Ferrara, 2000; Jenq et al., 2015). Conditioning regimen-induced epithelial atrophy, exacerbated by T cell-mediated damage, impairs barrier function across key mucosal cell types, including Paneth cells, Goblet cells, and intestinal stem cells (ISCs). Paneth cell depletion diminishes defensin production, decreasing selective pressure against non-commensals and enabling pathogenic bacterial overgrowth (Eriguchi et al., 2012; Levine et al., 2013). In patient biopsies, goblet cell loss was associated with severe GI-aGvHD and poor outcomes (Ara et al., 2020). These cells primarily secrete mucins to form a protective mucus barrier. Goblet cell loss—together with an expansion of mucolytic bacteria—erodes the mucus layer, fostering bacterial translocation and amplifying local and systemic inflammation (Hayase et al., 2022). In this study, MaaT013-treated germ-free mice exhibited an increase in colonic crypt depth concomitant with an increase in Goblet cell numbers, suggesting epithelial remodeling that bolsters barrier integrity and counters these pathological features.

MaaT013 demonstrated immunomodulatory capacity both *in vitro* and *in vivo*. In germ-free mice, MaaT013 administration was associated with transcriptomic changes consistent with modulation of immune homeostasis, upregulating pathways for balanced innate and adaptive responses—including B cell activation (BCR/CD22), effector cell activation and signaling (FcγR/FcεRI), and innate defenses (complement/phagocytosis)—while driving humoral reconstitution via IgA-related genes (*Igha, Jchain, Mzb1, Ccr10*) to bolster mucosal immunity. Concurrent elevations in regulatory markers (*Nfil3, Tigit, Arg2, Areg*), γδ T cell-epithelial interactions (*Btnl1*), and Paneth cell amplification (*Bhlha15*) promoted tissue repair, antimicrobial peptide secretion, and commensal preservation. This immune rebalancing, alongside epithelial strengthening (*Vill*) and metabolic adaptations (*Cyp2c55*), aligns with MaaT03's metabolite production (SCFAs, indoles) and *in vitro* findings, supporting a potential to reestablish gut-immune equilibrium post-HSCT.

MaaT03's immunomodulatory potential was further evidenced *in vitro*, promoting DCs toward a phenotype characterized by enhanced maturation markers (CD83), antigen presentation (HLA class II), co-stimulation (CD80, CD86, CD40), and regulatory molecules (CD39, PD-L1), alongside robust IL-10 production. This DC reprogramming preferentially amplifies Tregs, as evidenced by their markedly higher proliferation rates compared to conventional CD4^+^ T cells, coupled with elevated TGF-β1 and IL-10 secretion. Of note, human microbiota-reactive DP8α Tregs, whose murine counterpart are yet to be determined, were found to protect against aGvHD in a humanized NSG mouse model, underscoring the key role of microbiota/Treg interplay to limit aGvHD (Godefroy et al., 2024). Such selective Treg amplification (43% proliferation vs. 16% for conventional CD4^+^ T cells) mirrors mechanisms where commensal bacteria enhance DC cross-presentation of self-antigens to induce peripheral tolerance, potentially via SCFA-mediated HDAC inhibition or AhR signaling from indoles, thereby supporting a potential mechanistic link between microbiota-derived signals and immune regulation.

Using SPF NCG mice humanized with human PBMCs as a xenogeneic GvHD model, we demonstrate that the engraftment of beneficial bacteria contained in MaaT013 was associated with a temporal trend toward lower GvHD severity at intermediate time points. However, due to the profound immunodeficiency of these mice and the significant xenogeneic incompatibility between human PBMCs and mouse tissues, the rapid expansion of human PBMCs was accompanied by a dramatic increase in GvHD severity in both PBS and MaaT013-treated arms, resulting in the death of all mice at D31. As such, this mouse model of GvHD represents an aggravated form of the human disease (Wang et al., 2022). Consistent with biological data showing a persistent increase in human T cell numbers, the sudden increase of GvHD severity appears attributable to the inherent severity of the model itself (Mims et al., 2025). Notably, the sudden increase in GvHD severity in MaaT013-treated mice began prior to the final MaaT013 administration, indicating that cessation of treatment was not responsible for this escalation. Therefore, while the study supports the mechanism of action of MaaT013 through its ability to engraft in a xenogeneic mouse model of GvHD, the model represents an exacerbated form of the human disease and has limited clinical translatability (Ehx et al., 2024). Accordingly, the non-significant differences observed at D25 and D27 should be interpreted cautiously, as the small sample size and the rapid progression of severe xenogeneic GvHD in this model limit statistical power to detect modest treatment effects. In addition, further clinical studies are warranted to elucidate the respective roles of metabolites, antigen-presenting cells, and regulatory T cells in MaaT013-mediated GvHD control.

The proposed mechanism of action of MaaT013 in GI aGvHD is illustrated in Figure 6. The conditioning regimen administered before allo-HCT induces profound host tissue damage. Combined with the early administration of broad-spectrum antibiotics, this results in a profound disruption of gut microbiota, characterized by a loss of diversity and the overwhelming expansion of harmful bacteria. GI tissue damage leads to the release of damage-associated molecular patterns (DAMPs) and to the systemic exposure of PAMPs, microbial products normally sequestered in the gut lumen (Ferrara et al., 2009). These signals lead to the release of pro-inflammatory cytokines such as tumor necrosis factor alpha (TNFα), interleukin-1 beta (IL-1β) or type I interferon gamma (IFNγ) and to the activation of host antigen-presenting cells, enhancing their ability to present allo-antigens to donor T-cells. Host APCs then activate alloreactive mature donor CD4^+^ and CD8^+^ T cells. CD4^+^ T cells subsequently proliferate and differentiate into helper T cells (Th), such as Th1 and Th17 cells. Activated helper T cells secrete additional inflammatory cytokines such as IFNγ and IL-2, which further accelerates activation of CD8^+^ cytotoxic T lymphocytes (CTL) and natural killer (NK) cells. In turn, activated CTL, macrophages and NK cells release pro-inflammatory cytokines, causing tissue damage (Staffas et al., 2017) and resulting in aGvHD symptoms (Malard et al., 2023b). After MaaT013 administration, microbial engraftment is expected to increase the richness and diversity of GI microbiota, leading to the production of key anti-inflammatory metabolites (Riwes and Reddy, 2018) and to the restoration of GI barrier integrity. Restoration of GI barrier integrity is expected to limit systemic exposure of pathogenic microorganisms and/or proinflammatory signals, therefore limiting chronic inflammation and infections. Together with the production of anti-inflammatory metabolites, this may contribute to the expansion of regulatory T cells, which could potentially limit the activation of donor T cells by host APCs through the production of anti-inflammatory cytokines such as IL-10 and TGF-β (Pinzon-Leal et al., 2025).

**Figure 6:**
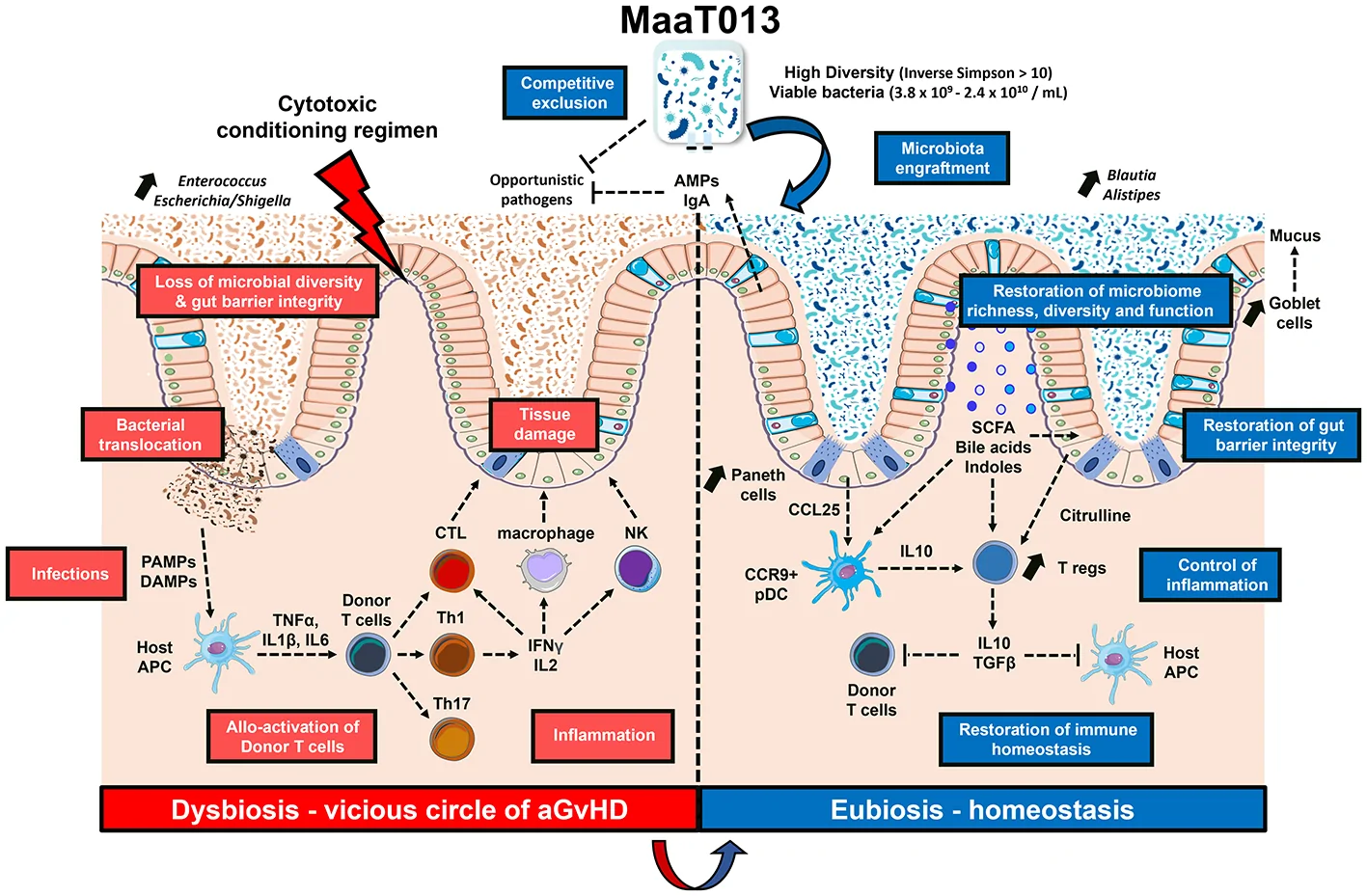
Proposed mechanism of action of MaaT013 in GI aGvHD. **Left** (Red): Physiopathology of GI aGvHD. **Right** (Blue): Restoration of gut eubiosis and control of GI aGvHD after MaaT013 administration. APC: antigen-presenting cells; PAMPs: pathogen-associated molecular patterns; DAMPs: damage-associated molecular patterns; Th: T helper; IL: interleukin; CTL: cytotoxic T lymphocytes; NK: natural killers; IFNγ: interferon gamma; AMPs: Antimicrobial peptides; IgA: immunoglobulin A; TNFα: tumor necrosis factor α, TGF-β: transforming growth factor beta, pDC: plasmacytoid dendritic cells; CCL25: C-C Motif Chemokine Ligand 25; CCR9: C-C Motif Chemokine Receptor 9; SCFA: Short Chain Fatty Acid. Adapted from Servier Medical Art (https://smart.servier.com), licensed under CC BY 4.0.

Altogether, these findings support the potential of MaaT013 in modulating biological processes involved in aGvHD pathogenesis. Through the restoration of microbial diversity, MaaT013 reintroduces key beneficial bacterial taxa with essential functional roles, including the induction of tolerogenic DCs and the production of anti-inflammatory metabolites, thus shifting the balance between alloreactive and regulatory T cells (Jarosch et al., 2023). These coordinated effects may contribute to reestablish intestinal barrier integrity and immune homeostasis and support MaaT013's potential to disrupt the self-sustaining inflammatory loop characteristic of GI aGvHD.

This study has several limitations that preclude direct extrapolation to human aGvHD patients. *In vitro* assays cannot capture interspecies microbial interactions, host-microbiota crosstalk, metabolic cross-feeding, or the dynamic intestinal environment influencing bacterial engraftment and function. Likewise, mouse models, although informative, differ substantially from humans, particularly in the context of microbiome-immune interactions. Germ-free models lack pre-existing microbiota and immune maturation, while humanized models are highly immunodeficient, suffer from limited microbial engraftment, and represent an aggravated xenogeneic form of GvHD which may mask potential therapeutic effects on survival. Importantly, germ-free mice were used as a reductionist model to determine whether MaaT013 reproducibly engrafts and to isolate biologically relevant host responses in a controlled microbiota-free environment. These experiments were not intended to assess comparative efficacy or establish therapeutic superiority relative to other microbiota-based interventions. In addition, the small sample sizes in the GvHD experiments limit statistical power and preclude firm conclusions regarding modest treatment effects. Accordingly, the present work should be interpreted as an exploratory mechanistic characterization of MaaT013 rather than a definitive demonstration of efficacy. Future studies will be required to compare MaaT013 with other microbiota-based interventions under standardized conditions. Approaches incorporating controlled microbial transfer, targeted metabolite supplementation, or host pathway perturbation in gnotobiotic or GvHD models will be instrumental in establishing causal relationships. Dedicated clinical studies will ultimately be required to confirm its proposed mechanism of action in aGvHD.

## Data Availability

The dataset associated with this study has been deposited in Figshare under DOI 10.6084/m9.figshare.31674022. The sequencing data that support the findings have been deposited in the NCBI SRA database under BioProject accession number PRJNA1435791.
